# Silencing circular RNA circ_0054537 and upregulating microRNA-640 suppress malignant progression of renal cell carcinoma via regulating neuronal pentraxin-2 (NPTX2)

**DOI:** 10.1080/21655979.2021.1984002

**Published:** 2021-10-21

**Authors:** Long Pei, Xianqiang Lv, Gaopei Jia, Xiaoliang Tan, Ming Li, Aili Zhang

**Affiliations:** Department of Urology, The Fourth Hospital of Hebei Medical University, Shijiazhuang City, Hebei Province, China

**Keywords:** Circ_0054537, miR-640, NPTX2, RCC

## Abstract

Hsa_circ_0054537 (circ_0054537) is a novel tumor-related circular RNA in renal cell carcinoma (RCC), and we intended to ascertain its dysregulation and functions in RCC malignant progression, as well as the underlying mechanism via serving as competing endogenous RNA (ceRNA). In this research, using real-time quantitative PCR, we found circ_0054537 was upregulated in RCC tissues and cells, and distributed throughout the cytoplasm. Then, functional effects of circ_0054537 in RCC were detected using cell counting kit-8, transwell, flow cytometry and glycolysis stress test and adenosine Triphosphate (ATP) assays. The results uncovered that circ_0054537 knockdown inhibited cell proliferation, migration, invasion, autophagy and glycolysis, but promoted apoptosis in RCC cells. Notably, circ_0054537 was identified as a ceRNA for microRNA (miR)-640, and miR-640 could target neuronal pentraxin-2 (NPTX2), as evidenced by dual-luciferase reporter assay and RNA immunoprecipitation assay. Besides, miR-640 downregulation or NPTX2 overexpression partly overturned the tumor suppressor function of circ_0054537 silence and miR-640 overexpression in RCC cells. Additionally, RCC cell growth *in vivo* was retarded by circ_0054537 silence. In conclusion, circ_0054537/miR-640/NPTX2 ceRNA pathway regulated RCC malignant progression *in vitro* and curbed RCC tumor growth *in vivo*, which could be a potential diagnosis and therapeutic target of RCC.

## Introduction

Renal cell carcinoma (RCC) is the most common cancer among all renal malignancies, and its incidence is still increasing worldwide in both men and women [1]. Histologically, RCC encompasses two major subtypes, including clear-cell RCC (ccRCC) accounting for about 70–80% and non-clear-cell RCC (non-ccRCC) accounting for about 20–30% [2,3]. The investigation of genetics and epigenetics of RCC are helpful to reveal molecular heterogeneity of RCC tumors and seek novel therapeutic target [4,5].

Circular RNAs (circRNAs) are produced by linking the 3ʹ and 5ʹ ends via covalent backsplicing. CircRNA expression profiles are altered in renal diseases including RCC [6], and many RCC-associated circRNAs have been uncovered to be important regulators in RCC carcinogenesis and progression [7]. Furthermore, several circRNAs are considered as promising biomarkers for the diagnosis and potential targets for RCC treatment [6,7]. Functioning as microRNAs (miRNAs) sponges or competing endogenous RNAs (ceRNAs), circRNAs can regulate and control downstream target genes expression in several human cancers [8]. Moreover, the biogenesis of miRNAs and their expression profiles, biological roles and clinical implications in RCC have been well-investigated to date [9].

Dysregulation of non-coding RNAs including circRNAs and miRNAs is a crucial part in epigenetics [10], which is emerging driving factor of ccRCC [11], and non-coding RNAs profiling is contribute to seeking novel target for early diagnosis and prognostic evaluation of RCC patients [12]. CircRNA hsa_circ_0054537 (circ_0054537) might be abnormally upregulated in a cohort of RCC cells and modulate RCC cell proliferation and apoptosis by sponging miRNA (miR)-130a-3p to regulate oncogene cMet [13]. However, the expression and role of circ_0054537 in diverse human cancers including RCC remained to be further discovered. Besides, miR-640 as a potential suppressor in breast cancer and hepatocellular carcinoma has been confirmed [14,15], and it may participate in the development of liver injury and intervertebral disc degeneration through serving as inflammatory actuator [16,17], as well as angiogenesis inhibitor [18,19]. Neuronal pentraxin 2 (NPTX2), which is the most frequently dysregulated gene in patient tumor tissues [20].

The aim of this study is to explore the dysregulation and functional roles of circ_0054537 in human RCC, and investigate the regulatory mechanism circ_0054537 in RCC malignant progression. Our research uncovered that circ_0054537 regulated RCC malignant progression *in vitro* and curbed RCC tumor growth *in vivo* by regulating miR-640/NPTX2 pathway, which may present a potential diagnosis and therapeutic target of RCC.

## Materials and methods

### Patient enrollment and tissue specimens

A total of 39 RCC specimens were surgically obtained from 39 patients diagnosed with primary RCC in The Fourth Hospital of Hebei Medical University. Their diagnoses were independently reviewed by two pathologists and classified by WHO criteria. Meanwhile, the corresponding para-carcinoma normal tissues were also obtained after written informed consents were obtained from these patients. The study was approved by the Ethics Committee of The Fourth Hospital of Hebei Medical University. Cancer tissues and normal tissues were stored for total RNA/protein isolation. The clinicopathological characteristics of patients with RCC are presented in Table 1.

**Table 1.** The association of circ_0054537 expression with clinicopathological characteristics of patients with RCC

Clinicopathologic features	Relative circ_0054537 level		P value
	High (%)	Low (%)	
Gender			0.7573
Male	13 (56.0)	12 (44.0)	
Female	8 (52.6)	6 (47.4)	
Age(years)			0.8428
≥60	10 (66.7)	8 (44.0)	
<60	11 (40.0)	10 (60.0)	
Tumor size (cm)			0.1382
≥3	8 (80.0)	3 (20.0)	
<3	13 (41.4)	15 (58.6)	
TNM stage			0.0204
I+ II	10 (34.8)	15 (65.2)	
III	11 (76.2)	3 (33.8)	
Metastasis			0.0281
No	12(43.8)	16 (56.2)	
Yes	9 (83.3)	2 (16.7)	
Histology			0.7079
Papillary	14 (51.9)	13 (48.1)	
Clear Cell	7 (58.3)	5 (41.7)	

### Cell culture and cell transfection

Human RCC 786-O (no. 60,243) and A498 (no. 60,241) cell lines were originally from Bioresource Collection and Research Center (Taiwan, China), as well as human kidney HK-2 (no. 60,097) cells. A498 cells were cultured in the Minimum Eagle’s Medium (M4655; Sigma-Aldrich, St Louis, MO, USA), 786-O cells were in Roswell Park Memorial Institute-1640 medium (R8758; Sigma-Aldrich), and HK-2 cells were in Defined Keratinocyte-Serum Free Medium Kit (10,744,019; GIBCO, Grand Island, NY, USA). All cells were incubated in 90% corresponding medium and 10% Fetal Bovine Serum (10,100,147; GIBCO) at 37°C with 5% CO_2_.

For transfection, 786-O and A498 cells were seeded in 6-well plate overnight and then transfected with small interfering RNAs (siRNAs), short hairpin RNAs (shRNAs), miRNA mimic, miRNA inhibitors, and plasmids using Lipofectamine RNAiMAX (Invitrogen, Carlsbad, CA, USA). The siRNAs targeting circ_0054537 (si-circ_0054537^#1,#2^ and ^#3^) are presented in Table 2, as well as the scrambled control siRNA (si-con). The sequences of si-circ_0054537^#1^ were inserted into pGPH1/Neo plasmid (GenePharma, Shanghai, China) to construct sh-circ_0054537 plasmid. Similarly, sh-con plasmid was obtained. pcDNA (zeo+) plasmid (BioVector Science Lab, Beijing, China) was used to construct pcDNA-NPTX2 overexpression plasmid (NPTX2). miR-640 was exogenously overexpressed and inhibited using miR-640 mimic and miR-640 inhibitor (in-miR-640). The corresponding controls also included miR-con mimic and in-miR-con. Transfected cells were harvested at 30 h for further functional analysis.

**Table 2.** The sequences of si (sh)-RNAs and RT-qPCR primers

Name	Sequence (5ʹ-3ʹ)
si-circ_0054537^#1^ sense	GGAUUCUUUUUAACAGGUGGGdTdT
si-circ_0054537^#1^ antisense	CCCACCUGUUAAAAAGAAUCCdTdT
si-circ_0054537^#2^ sense	AUUCUUUUUAACAGGUGGGGGdTdT
si-circ_0054537^#2^ antisense	CCCCCACCUGUUAAAAAGAATdTdT
si-circ_0054537^#3^ sense	GAUUCUUUUUAACAGGUGGGGdTdT
si-circ_0054537^#3^ antisense	CCCCACCUGUUAAAAAGAAUCdTdT
sh-circ_0054537 sense	GGAUUCUUUUUAACAGGUGGG
sh-circ_0054537 antisense	CCCACCUGUUAAAAAGAAUCC
si (sh)-NC sense	CCAUAUCUGCGAUUUCUGUUGAUAA
si (sh)-NC antisense	UUAUCAACAGAAAUCGCAGAUAUGG
circ_0054537 forward primer	GTCGGCATACACAGGCGTAA
circ_0054537 reverse primer	TCCATGAGCCCAGGGACA
PSME4 forward primer	CGCAGGGCGACGAAGAC
PSME4 reverse primer	ATCTCCTTCTGCGGGACGAA
miR-640 forward primer	GCCCCTGCAGAGCACTGCGG
miR-640 reverse primer	GGCCACCCGGCGGCCGGCAA
NPTX2 forward primer	TCTTCCCGTCTGAAGAACGC
NPTX2 reverse primer	CGGAGATCACAGCCCTTCTC
β-actin forward primer	TCACCATGGATGATGATATCGC
β-actin reverse primer	ATAGGAATCCTTCTGACCCATGC
U6 forward primer	CCGTATGACCTCCTTCCACAGA
U6 reverse primer	TCTGTCCACCTCTGAAACCAGG

### Subcellular localization and structural character of circ_0054537

Nuclear/Cytosol Fractionation Kit (K266; Biovision, San Francisco, CA, USA) was used to separate nuclear extract and cytoplasmic fraction of 786-O and A498 cells, and the nuclear and cytoplasmic RNAs were isolated in TRIzol reagent (Invitrogen). Then, the expression of circ_0054537, U6 nucleolus small RNA (U6) and β-actin was detected by real-time quantitative PCR (RT-qPCR) and compared between cytoplasm and nucleus. Total RNAs in 786-O and A498 cells were also secreted using TRIzol reagent (Invitrogen), and 2 μg aliquot of total RNAs were treated with 5 U RNase R (Geneseed Biotech, Guangzhou, China) for 30 min at 37°C. Then, to compare the expression stability of circRNA and its host gene, the expression of circ_0054537 and mRNA of proteasome activator subunit 4 (PSME4; the host gene of hsa_circ_0054537 = hsa_circPSME4_001) under RNase R treatment (RNase R^+^) or RNase R untreatment (RNase R^−^) was detected by RT-qPCR.

### RT-qPCR and western blotting

RNAs were reverse transcribed into cDNA using miScript Reverse Transcription Kit (Qiagen, Dusseldorf, Germany), and RT-qPCR was performed with the diluted cDNA, special primers and Fast SYBR™ Green Master Mix (Applied Biosystems). Primers for circ_0054537, PSME4, miR-640, NPTX2, U6, and β-actin were designed using Primer3 software and synthesized by GENEWIZ (Beijing, China). The primer sequences are presented in Table 2. The melting curve was drawn to ensure primer specificity, and RNA expression levels were determined by cycle threshold (CT) method [21]. The relative RNA expression was normalized to U6 or β-actin.

Total proteins were isolated from tissues and cells in RIPA reagent (Sigma-Aldrich), and 20 μg aliquot of that were subjected to western blotting assay to evaluate expression of special proteins related to apoptosis (Bcl2-associated X protein [Bax] [22]), autophagy (LC3 [23]) and metastasis (matrix metalloproteinase 9 [MMP-9] [24]). The primary antibodies including anti-LC3 (4600-1-AP, 1:2,500; Proteintech, Wuhan, China), anti-NPTX2 (10,889-1-AP, 1:1,000; Proteintech), anti-MMP9 (ab228402; 1:5,000; Abcam, Shanghai, China), anti-Bax (ab216494; 1:500; Abcam), and anti-β-actin (20,536-1-AP, 1:5,000; Proteintech) were incubated overnight at 4°C, and secondary antibody anti-Rabbit IgG (SA00001-2, 1:10,000, Proteintech) was incubated for 2 h at 25°C. Eventually, proteins were detected using ECL Western Blotting Substrate (Pierce, Rockford, IL, USA). Protein expression was determined by gray density of blots analyzed on Image J software (NIH, Bethesda, MD, USA), and relative protein expression was normalized to β-actin.

### RNA fluorescence in situ hybridization (RNA-FISH)

Biotin-labeled oligonucleotides targeting circ_0054537 were served as circ_0054537 probe sequence. Cells were treated as previously reported [25]. In brief, dehydrated cell suspension was hybridized with probes (green), and sealed with parafilm containing DAPI (nuclei dye: blue). Fluorescence in cells was observed under a fluorescence microscopy.

### Cell counting kit-8 (CCK-8) assay, flow cytometry (FCM) and transwell assays

Cell proliferation, apoptosis and migration&invasion of 786-O and A498 cells were respectively measured by CCK-8 reagent (Vazyme, Nanjing, China), Annexin-V-fluorescein isothiocyanate (FITC)/propidium iodide (PI) apoptosis detection kit (Vazyme), and Transwell chambers (Corning, Cambridge, UK) without and without Matrigel (BD Biosciences; San Jose, CA, USA). The detail methods were provided in the Supplementary file.

### Real-time measurement of extracellular acidification rate (ECAR)

Lactate excretion was also accompanied by extracellular acidification, and ECAR is known to correlate with the glycolytic activity [26]. The XF96 extracellular flux analyzer (Seahorse Bioscience, North Billerica, MA) was used to measure ECAR according to the manufacturer’s instructions. 1 × 10^4^786-O and A498 cells were incubated with un-buffered medium in sequence adding 10 mM glucose, 1 mM oligomycin (oxidative phosphorylation inhibitor) and 80 mM 2-deoxyglucose (2-DG; glycolytic inhibitor). Lastly, cell number was used to normalize the final ECAR values and ECAR was shown in mpH/minute.

### Adenosine triphosphate (ATP) detection

Intracellular ATP production detection was performed by using the ATP Assay Kit (Beyotime, Shanghai, China). Briefly, 786-O and A498 cells were treated for 6 h and incubated in Lysis Buffer for 10 min at 4°C. The supernatant was collected by centrifuging at 12,000 × g at 4°C for 10 min and reacted with ATP Assay Working Solution. Then, relative light units (RLU) values were analyzed by a luminometer (Promega, Madison, WI, USA), and relative ATP level was normalized to corresponding control group.

### Bioinformatics database analysis

Gene Expression Omnibus database (accession GSE61741: https://www.ncbi.nlm.nih.gov/geo/query/acc.cgi?acc=GSE61741) was used to search abnormally downregulated miRNAs in renal cancer patients’ blood and normal controls’ blood. GEPIA database (http://gepia.cancer-pku.cn/detail.php?gene=NPTX2&clicktag=boxplot) was used to measure NPTX2 expression in tissues of kidney renal clear cell carcinoma (KIRC, also named as ccRCC) and normal tissues. The potential binding sites between miR-640 and circ_0054537 or NPTX2 were predicted on Circular RNA Interactome database (https://circinteractome.nia.nih.gov/mirna_target_sites.html) and microT CDS database (http://diana.imis.athena-innovation.gr/DianaTools/index.php?r=microT_CDS/index). To analyze the clinical value of circ_0054537, these RCC patients were divided into two groups: High circ_0054537 (n = 21) and Low circ_0054537 (n = 18) according to the average of circ_0054537 level in all these 39 RCC tumor tissues, and the correlation between circ_0054537 level and clinicopathological characteristics is shown in Table 1.

### Dual-luciferase reporter assay and RNA immunoprecipitation (RIP)

The circ_0054537 fragments and 3ʹ-untranslated region (3ʹUTR) of NPTX2 (NPTX2-3ʹUTR) that incorporated specific miR-640-binding sites were separately cloned into the pmirGLO Dual-Luciferase miRNA Target Expression Vector (Promega) to construct pmirGLO vectors carrying wild type (WT) of circ_0054537 (circ_0054537-WT) and NPTX2-3ʹUTR-WT. Similarly, the specific miR-640-binding sites were mutated, and pmirGLO vectors carrying mutant of circ_0054537 (circ_0054537-MUT) and NPTX2-3ʹUTR-MUT were constructed. 786-O and A498 cells were co-transfected with above pmirGLO vectors, pRL-TK vector (Promega), and either miR-640 mimic or miR-con mimic.

Cell lysate was collected in RIPA reagent (Sigma-Aldrich) supplemented with 200 U/mL RNase Inhibitor (Sigma-Aldrich). Protein G Sepharose 4 Fast Flow bead slurry (GE Healthcare, Beijing, China) was pre-coated with antibody against human Ago2 (anti-Ago2; ab32381, 1:50; Abcam) or the negative control anti-IgG (ab109489, 1:100; Abcam). The supernatant of cell lysate was incubated overnight with above Sepharose beads at 4°C. The immunoprecipitated complex was incubated with 10 mg/mL proteinase K (Sigma-Aldrich) for 30 min at 37°C, then RNAs bond to Ago2 or IgG were isolated by TRIzol reagent (Invitrogen), and expression of circ_0054537, miR-640 and NPTX2 mRNA was detected by RT-qPCR.

### Biotin-labeled miRNA capture assay

786-O and A498 cells were transfected with biotinylated miR-640 (bio-miR-640) or bio-miR-con (GenePharma), and cell lysates were incubated with pre-blocked streptavidin magnetic beads. Then, the abundance of mRNAs of interest in the elution contents from the beads was determined by RT-qPCR.

### Xenograft model

Three groups of five nude mice (BALB/c/nu/nu, male, Beijing Vital River Laboratory Animal Technology, Beijing, China) each were subcutaneously injected with A498 cells or A498 cells stably transfected with sh-circ_0054537 vector or sh-con. In brief, 2 × 10^6^ cells in 100 μL normal saline were used to cell inoculation, and equal volume of normal saline was used in Mock group. The xenograft tumors were examined every week for 4 weeks, and the larger and shorter sizes (*l* and *s*) in perpendicular indexes were measured by vernier caliper. The weight of tumors were examined on electronic scales on the last day after the mice were euthanized. The tumor volume was calculated as 0.5 × *l*× *s*^2^. The tumor tissues were restored for further total RNA/protein isolation or immunohistochemistry of paraffin embedding (IHC-P) [27]. Antibodies were MMP-9 (ab228402; 1:5,000; Abcam), Bax (ab216494; 1:500; Abcam) and ki-67 (ab15580; 1:50; Abcam). Animal experiments were approved by the Ethics Committee of The Fourth Hospital of Hebei Medical University. All the animals were subjected to humanitarian care, and all experiments were performed in accordance with the Guide for the Care and Use of Laboratory Animals (Ministry of Science and Technology of China, 2006).

### Statistical analysis and correlation analysis

The quantitative data were shown as mean±standard deviation and compared with Student’s test or one way analysis of variance on GraphPad prism software (GraphPad, San Diego, CA, USA). Pearson’s correlation analysis was used to analyze the mutual correlations among circ_0054537, miR-640 and NPTX2 mRNA in RCC tissues. In all cases, *P* < 0.05 was considered statistically significant and signed as *.

## Results

### Circ_0054537 was an upregulated circRNA in RCC patients and cells

**Figure 1.**
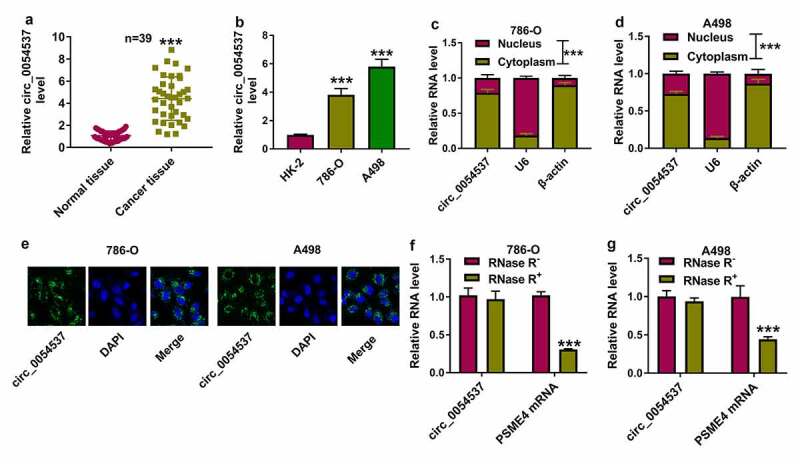
Circ_0054537 was an upregulated circRNA in RCC patients and cells. (a-d) RT-qPCR compared (a) circ_0054537 level between cancer tissues and normal tissues from RCC patients (n = 39), (b) circ_0054537 level between cancer cells (786-O and A498) and normal cells (HK-2), (c, d) circ_0054537, U6 and β-actin levels between nucleus and cytoplasm of 786-O and A498 cells. (e) RNA-FISH detected subcellular location of circ_0054537 in 786-O and A498 cells. Nucleus was stained with DAPI. (f, g) circ_0054537 and PSME4 mRNA levels between RNase R^+^ and RNase R^−^ groups in786-O and A498 cells. ***P < 0.001

Expression of circ_0054537 in RCC was firstly detected. According to circBase database (http://www.circbase.org/cgi-bin/simplesearch.cgi), circ_0054537 was derived from PSME4 gene (NM_014614) with a spliced sequence length of 7099. RT-qPCR analysis detected higher level of circ_0054537 in cancer tissues than paired normal tissues from RCC patients (Figure 1(a)). Similarly, circ_0054537 expression was increased in RCC cell lines (786-O and A498) comparing to normal renal HK-2 cells (Figure 1(b)). Moreover, subcellular localization of circ_0054537 was detected. Consequently, circ_0054537 was mainly localized in the cytoplasm (Figure 1(c) and 1D), and RNA-FISH further confirmed this result (Figure 1(e)). Additionally, comparing to the linear transcript PSME4 mRNA, circ_0054537 was more resistant to RNase R digestion, as indicated by the unchanged level of circ_0054537 and the reduced PSME4 level in RNase R^+^ group (Figure 1(f) and (g)). Altogether, circ_0054537 was a stably upregulated circRNA in RCC.

### *Circ_0054537 deletion constrained RCC cell malignant progression* in vitro

**Figure 2.**
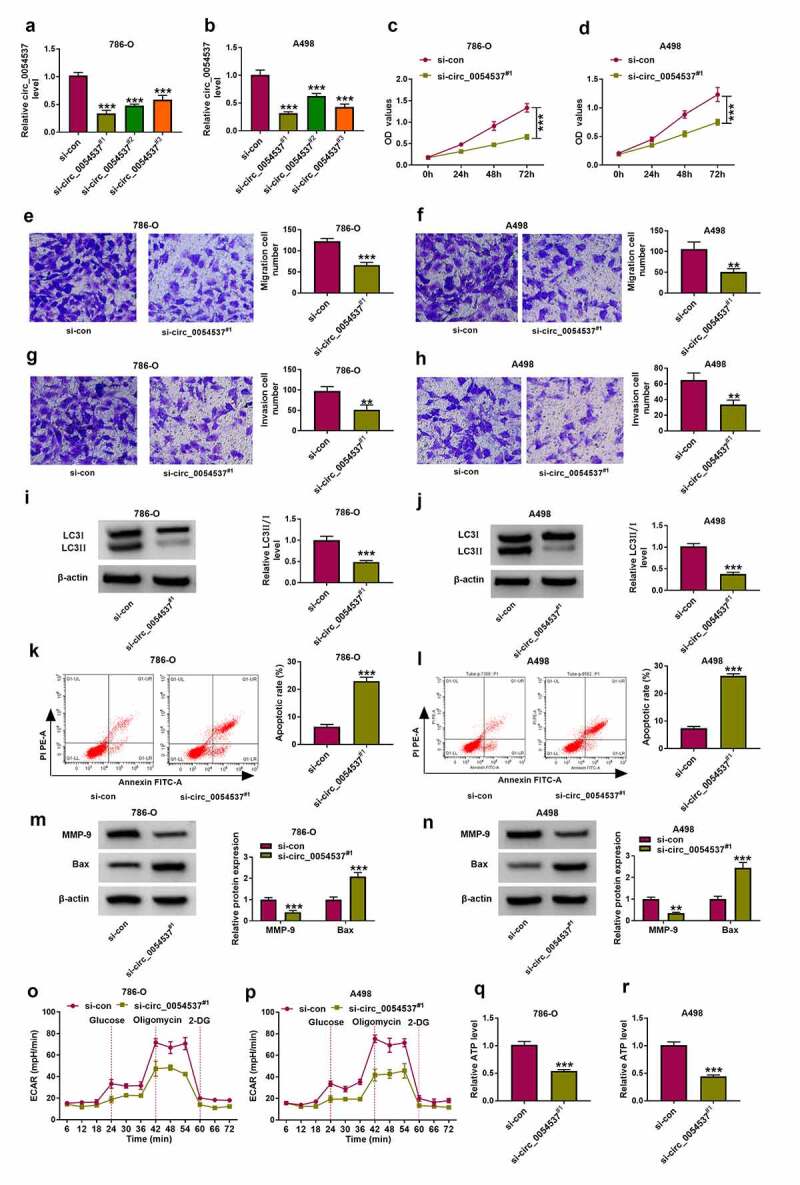
Circ_0054537 deletion boycotted cancer properties of RCC cells *in vitro*. (a, b) RT-qPCR compared circ_0054537 level between si-con transfection and si-circ_0054537 (^#1, #2^ and ^#3^) transfection in 786-O and A498 cells. (c, d) CCK-8 assay compared OD values, (e, f) transwell assays compared migration cell number, (g, h) transwell assays with Matrigel compared invasion cell number, (I, J, M, N) western blotting compared protein levels of LC3 II/I, MMP-9 and Bax, (k, l) FCM compared apoptotic rate (%), (o, p) XF96 extracellular flux analyzer compared ECAR (mpH/minute), and (q, r) ATP assay Kit compared ATP level between si-con transfection and si-circ_0054537^#1^ transfection in 786-O and A498 cells. **P < 0.01 and ***P < 0.001

Then, functional effects of circ_0054537 were measured in RCC cells. Three siRNAs targeting circ_0054537 were used to silence circ_0054537 expression in 786-O and A498 cells, and si-circ_0054537^#1^ was the most effective one (Figure 2(a) and (b)). si-circ_0054537^#1^ transfection inhibited the proliferation of 786-O and A498 cells, as evidenced by the lower OD values in si-circ_0054537#1 transfected cells (Figure 2(c) and d)). Transwell assays showed a reduction of migration cell number and invasive cell number in si-circ_0054537^#1^-transfected 786-O and A498 cells (Figure 2(e–h)), accompanied with lower expression of MMP-9 (Figure 2(m) and (n)). The expression of autophagy marker LC3 was measured in siRNAs-transfected 786-O and A498 cells by western blotting, and LC3 II/I level was diminished with si-circ_0054537^#1^ transfection than si-NC transfection (Figure 2(i) and (j)). Meanwhile, apoptotic rate determined by FCM method was highly induced in si-circ_0054537^#1^ group (Figure 2(k) and (l)), paralleled with higher Bax expression (Figure 2(m) and (n)). Metabolic reprograming and epigenetic alterations were recognized cancer hallmarks, and their interaction was still in its infancy concerning RCC [28]. ECAR and ATP level in 786-O and A498 cells were reduced by si-circ_0054537^#1^ transfection instead of si-NC transfection (Figure 2(o–r)). These results demonstrated that circ_0054537 knockdown inhibited RCC cell malignant progression by regulating cell proliferation, migration, invasion, autophagy, glycolysis, and apoptosis.

### miR-640 was downregulated in RCC, and was negatively regulated by circ_0054537

**Figure 3.**
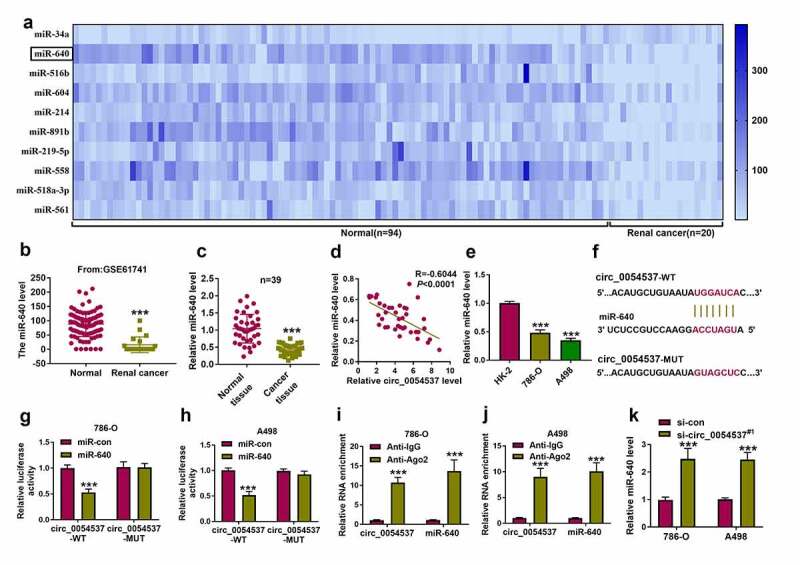
miR-640 was downregulated in RCC and correlated with circ_0054537 via targeting. (a, b) GSE61741 compared miRNAs levels including miR-640 in peripheral bloods between renal cancer patients and normal controls. (c, e) RT-qPCR detected miR-640 level in 39 RCC patients’ tissues and cells (HK-2, 786-O and A498). (d) Pearson’s correlation analysis analyzed the correlation between circ_0054537 and miR-640 levels in RCC tissues. (f) The predicted miR-640-binding sites in circ_0054537-WT were mutated. (g, h) Dual-luciferase reporter assay system compared luciferase activity of circ_0054537-WT/MUT vectors between miR-con mimic (miR-con) transfection and miR-640 mimic (miR-640) transfection in 786-O and A498 cells. (i, j) RIP assay detected RNA enrichment of circ_0054537 and miR-640 between anti-Ago2-mediated complex and anti-IgG-mediated complex. (k) RT-qPCR compared miR-640 level between si-con-transfected 786-O and A498 cells and si-circ_0054537^#1^-transfected above cells. ***P < 0.001

Accumulating evidence has suggested that miRNA mediated several biological pathways to participate in the regulation of RCC progression [29]. The microarray data obtained from Gene Expression Omnibus (GSE61741) showed several abnormally expressed miRNAs in renal cancer patients, and the top 10 downregulated miRNAs in 20 renal cancer bloods were exhibited (Figure 3(a) and Supplementary Table S1). And miR-640 was the second most downregulated miRNA in RCC bloods after miR-34a (Figure 3(a) and Supplementary Table S1). Given that circ_0054537 may function as miRNA sponge in RCC cells, we further predicted the potential miRNAs that targeted by circ_0054537. Circinteractome database showed that circ_0054537 contained the complementary binding sites of miR-640, but not miR-34a. Therefore, miR-640 was eventually selected for further investigation. Consistent with the dramatically decreased expression level of miR-640 in RCC bloods (Figure 3(b)), miR-640 was also downregulated in RCC tumor tissues (Figure 3(c)). Besides, the expression of miR-640 was negatively correlated with circ_0054537 in RCC tissues (Figure 3(d)). Additionally, miR-640 was downregulated in human RCC cell lines as well (Figure 3(e)). To verify the correlation between circ_0054537 and miR-640, dual-luciferase reporter assay was conducted. As displayed in Figure 3(f), circ_0054537 sequence contained the wild type (WT) or mutant type (MUT) miR-640 binding sites were cloned into the pmirGLO vector. And the results disclosed that transfection of miR-640 mimic significantly inhibited the luciferase activity of circ_0054537-WT group, but not circ_0054537-MUT group (Figure 3(g) and (h)). Ago2-RIP assay represented that circ_0054537 and miR-640 were significantly enriched in cells incubated with Ago2 antibody in contrast with that in IgG group (Figure 3(i) and (j)). In addition, miR-640 expression level was elevated with si-circ_0054537^#1^ transfection in 786-O and A498 cells (Figure 3(k)). These data demonstrated that miR-640 was downregulated in RCC and its expression was correlated with circ_0054537.

### Circ_0054537 regulating RCC cell malignant progression by sponging miR-640

**Figure 4.**
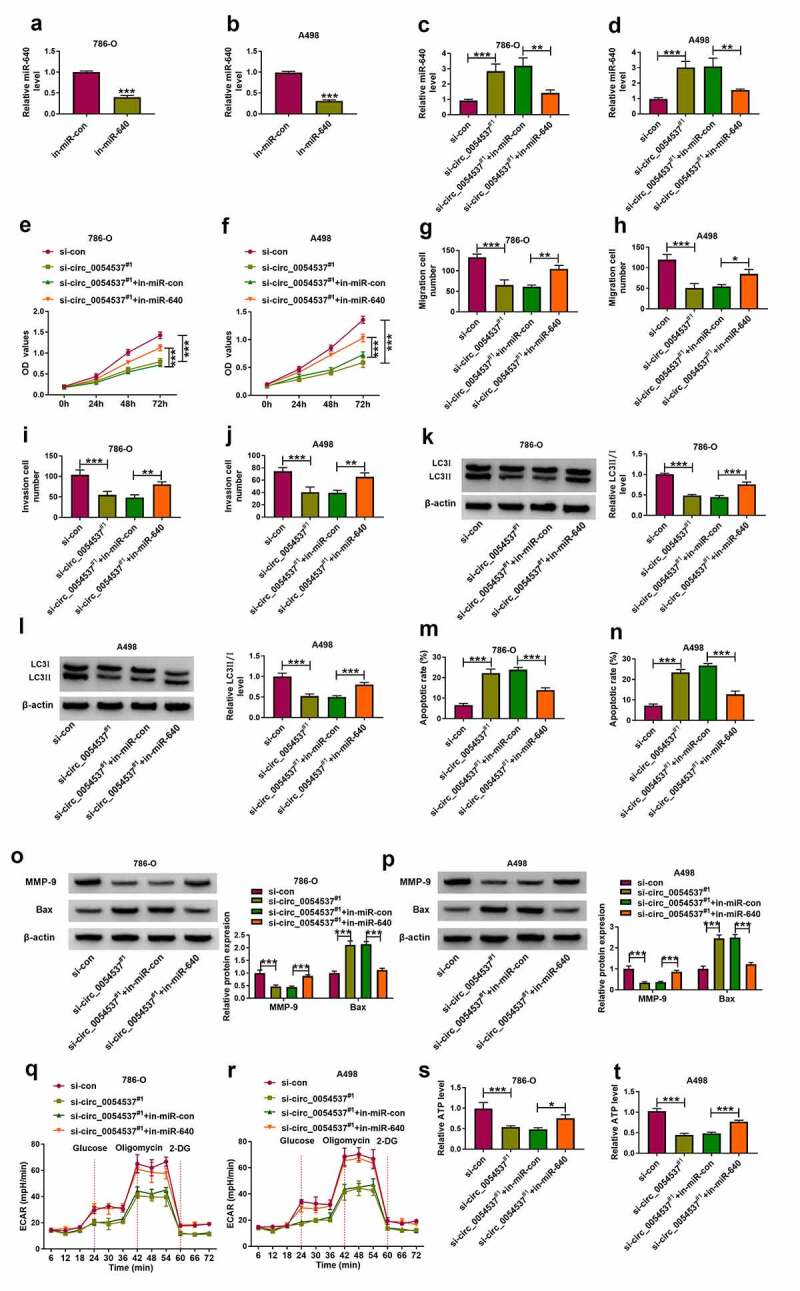
miR-640 inhibition promoted cancer properties of RCC cells with circ_0054537 deletion *in vitro*. (a-d) RT-qPCR compared miR-640 level in 786-O and A498 cells transfected with in-miR-con, in-miR-640, si-con, or si-circ_0054537^#1^, and co-transfected with si-circ_0054537^#1^ and in-miR-con or in-miR-640. (e, f) CCK-8 assay compared OD values, (g-j) transwell assays compared migration cell number and invasion cell number, (K, L, O, P) western blotting compared LC3 II/I, MMP-9 and Bax protein levels, (m, n) FCM compared apoptotic rate (%), (q, r) XF96 extracellular flux analyzer compared ECAR (mpH/minute), and (s, t) ATP assay Kit compared ATP levels between transfections of si-con and si-circ_0054537^#1^, and co-transfections of si-circ_0054537^#1^ with in-miR-con or in-miR-640 in 786-O and A498 cells. *P < 0.05, **P < 0.01 and ***P < 0.001

To further confirm the interaction between circ_0054537 and miR-640 in regulating RCC cell progression, rescue experiments were carried out. The in-miR-640 transfection resulted in the decrease of miR-640 in 786-O and A498 cells (Figure 4(a) and (b)), and upregulation of miR-640 in circ_0054537-silenced cells was diminished by additionally transfecting in-miR-640 (Figure 4(c) and (d)). OD values of 786-O and A498 cells were lowered by circ_0054537 knockdown, and this suppression effect could be attenuated by miR-640 downregulation (Figure 4(e) and (f)). The reduction of migration cell number and invasion cell number (Figure 4(g–j)), as well as MMP-9 expression (Figure 4(o) and (p)) mediated by circ_0054537 knockdown was also consistently rescued by the addition of in-miR-640. Furthermore, transfection of in-miR-640 partly overturned the effect of si-circ_0054537 on cell autophagy (Figure 4(k) and (l)) and apoptosis (Figure 4(m–p)). Meanwhile, the suppression effects of circ_0054537-depleted on ECAR and ATP level in 786-O and A498 cells were relieved by transfection with in-miR-640 (Figure 4(q–t)). Collectively, circ_0054537 acted as miR-640 sponge to regulate RCC cancer progression.

### NPTX2 was upregulated in RCC and targeted by miR-640

**Figure 5.**
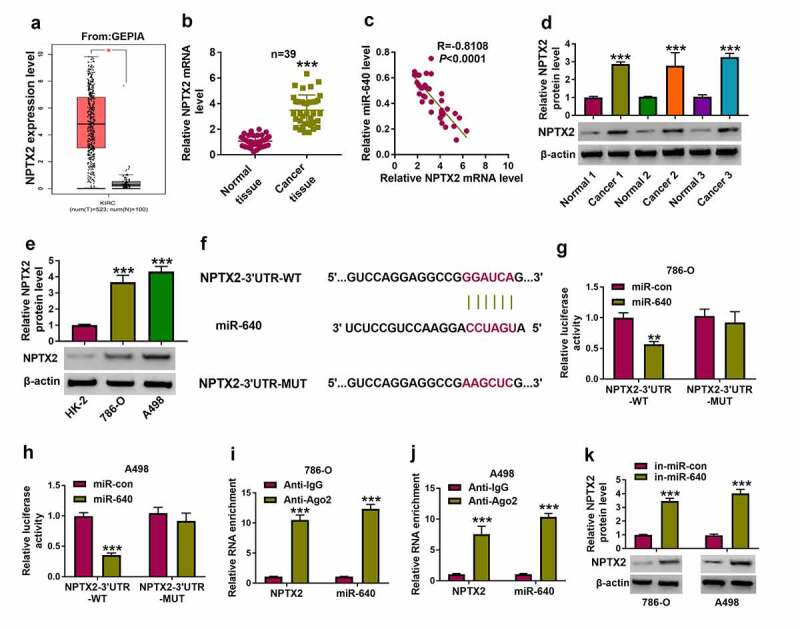
NPTX2 was upregulated in RCC and targeted by miR-640. (a) GEPIA showed NPTX2 expression on box plots in kidney renal clear cell carcinoma (KIRC, also named as ccRCC) tumor tissues and normal tissues. (b) RT-qPCR detected NPTX2 mRNA level in tissues from 39 RCC patients. (c) Pearson’s correlation analysis analyzed the correlation between miR-640 and NPTX2 mRNA levels in RCC tissues. (d, e) Western blotting detected PTX2 protein level in 39 RCC patients’ tissues and cells (HK-2, 786-O and A498). (f) The predicted miR-640-binding sites in NPTX2-3ʹUTR-WT were mutated. (g, h) Dual-luciferase reporter assay compared luciferase activity of NPTX2-3ʹUTR-WT/MUT vectors between miR-con transfection and miR-640 transfection in 786-O and A498 cells. (i, j) RIP assay detected RNA enrichment of miR-640 and NPTX2 mRNA between anti-Ago2-mediated complex and anti-IgG-mediated complex. (k) Western blotting compared NPTX2 protein level between in-miR-con transfection and in-miR-640 transfection in 786-O and A498 cells. **P < 0.01 and ***P < 0.001

Furthermore, functional genes directly regulated by circ_0054537/miR-640 were identified. Among the predicted results in microT CDS database, we preliminarily selected five mRNAs (NPTX2, Spinophilin [SPN], lysine demethylase 3A [KDM3A], SRY-box transcription factor 4 [SOX4], PR/SET domain 14 [PRDM14]) that had been demonstrated to be upregulated in RCC [20,30–33], and then their interactions with miR-640 were examined by biotin RNA pull down assay (Supplementary Figure S1A and S1B). Consequently, miR-640 was chosen as the optimal candidate target gene for miR-640 due to its highest enrichment in cells incubated with bio-miR-640. According to GEPIA data, NPTX2 expression level was higher in KIRC tumor tissues (Figure 5(a)), and RT-qPCR and western blotting data also displayed an upregulation of NPTX2 level in these 39 RCC cancer tissues (Figure 5(b) and (d)). Moreover, NPTX2 mRNA level was inversely correlated with miR-640 in RCC tissues (Figure 5(c)). In addition, NPTX2 protein level in 786-O and A498 cells was higher than that in HK-2 cells (Figure 5(e)). Unlike NPTX2-3ʹUTR-WT report vectors, NPTX2-3ʹUTR-MUT report vectors carrying mutant type miR-640 binding sites, and showed unresponsive to miR-640 ectopic expression in 786-O and A498 cells (Figure 5(f–h)). RIP assay declared a significant enrichment of miR-640 and NPTX2 levels in anti-Ago2-mediated precipitated complex (Figure 5(i) and (j)). Besides, miR-640 inhibition induced the increase of NPT2 protein level in 786-O and A498 cells (Figure 5(k)). These outcomes determined the target relationship between miR-640 and NPTX2 in RCC cells.

### Overexpressed miR-640 counteracted RCC progression by inhibiting NPTX2

**Figure 6.**
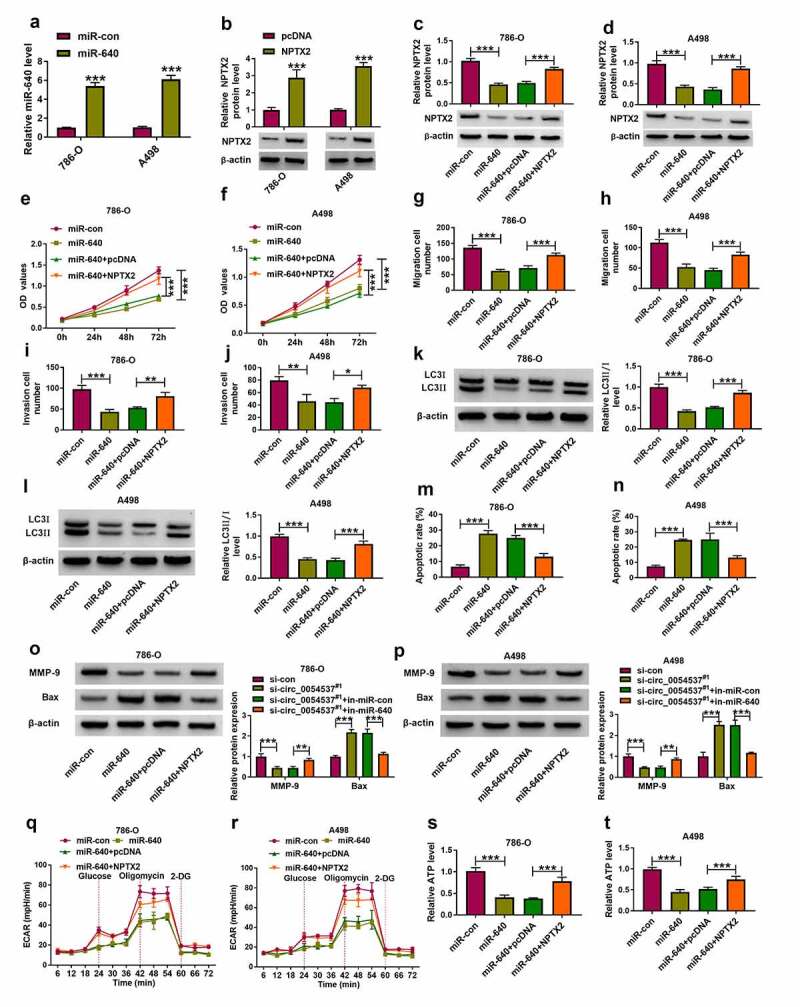
NPTX2 upregulation abrogated the tumor-suppressive role of miR-640 in RCC cells *in vitro*. (a) RT-qPCR detected miR-640 level and (b-d) western blotting detected PTX2 protein level in 786-O and A498 cells transfected with miR-con, miR-640, pcDNA vector, or pcDNA-NPTX2 (NPTX2) vector, and co-transfected with miR-640 and pcDNA or NPTX2 vector. (e, f) CCK-8 assay compared OD values, (g-j) transwell assays compared migration cell number and invasion cell number, (K, L, O, P) western blotting compared LC3 II/I protein level, (m, n) FCM compared apoptotic rate (%), (q, r) XF96 extracellular flux analyzer compared ECAR (mpH/minute), and (s, t) ATP assay Kit compared ATP levels between transfections of si-con and si-circ_0054537^#1^, and co-transfections of si-circ_0054537^#1^ with in-miR-con or in-miR-640 in 786-O and A498 cells. *P < 0.05, **P < 0.01 and ***P < 0.001

Besides, role of miR-640 and its interaction with NPTX2 were further confirmed in RCC cells. Transfection efficiencies of miR-640 mimic and NPTX2 were confirmed by RT-qPCR (Figure 6(a) and (b)), and overexpression of NPTX2 partly overturned the promotion effect of miR-640 on NPTX2 protein level (Figure 6(b–d)). With miR-640 upregulation, cell proliferation, migration and invasion of 786-O and A498 cells were diminished, as evidenced by the decreased OD values (Figure 6(e) and (f)), migration number and invasion cell number (Figure 6(g–j)), as well as MMP-9 expression (Figure 6(o) and (p). Autophagy-related marker LC3II/I level was inhibited in miR-640 mimic-transfected 786-O and A498 cells (Figure 6(k) and (l)), whereas the apoptotic rate and Bax expression were consistently elevated (Figure 6(m–p)). Overexpression miR-640 also declined ECAR and ATP level in 786-O and A498 cells (Figure 6(q–t)). Notably, transfection of pcDNA-NPTX2 plasmid in miR-640-overexpressed 786-O and A498 cells could improve the proliferation, migration, invasion and autophagy, but attenuate apoptosis (Figure 6(e–t)). These data indicated the tumor-suppressive effect of miR-640 in RCC cells, and uncovered that miR-640 could exert its function by interacting with NPTX2.

### Circ_0054537 mediated the expression of NPTX2 in RCC via miR-640

**Figure 7.**
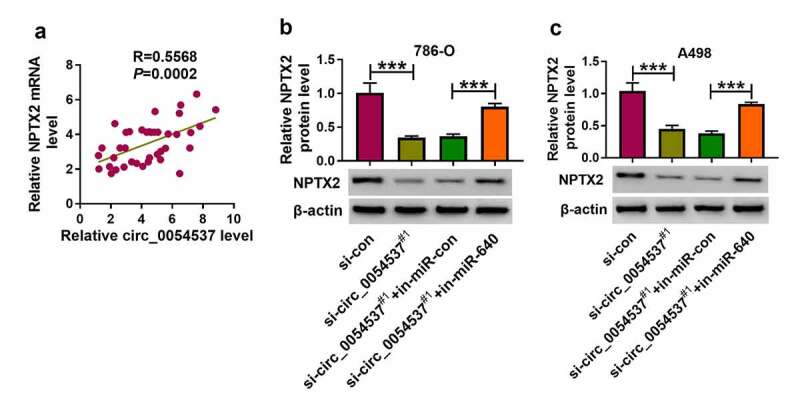
NPTX2 expression was correlated with circ_0054537 in RCC via miR-640. (a) Pearson’s correlation analysis analyzed the correlation between circ_0054537 and NPTX2 mRNA levels in RCC tissues. (b, c) Western blotting detected PTX2 protein level in 786-O and A498 cells transfected with si-con or si-circ_0054537#1, and co-transfected with si-circ_0054537^#1^ and in-miR-con or in-miR-640. ***P < 0.001

Notably, the correlation between circ_0054537 and NPTX2 via miR-640 was testified. According to Pearson’s correlation analysis, NPTX2 mRNA expression also positively correlated with circ_0054537 in RCC patients’ tissues (Figure 7(a)). Besides, NPTX2 protein level was inhibited in circ_0054537-silenced 786-O and A498 cells, and co-transfection of si-circ_0054537^#1^ and in-miR-640 could cancel this effect (Figure 7(b) and (c)). These outcomes uncovered the association among circ_0054537, miR-640 and NPTX2 in RCC.

### *Circ_0054537 silence retarded RCC tumor growth* in vivo *by regulating miR-640/NPTX2 axis*

**Figure 8.**
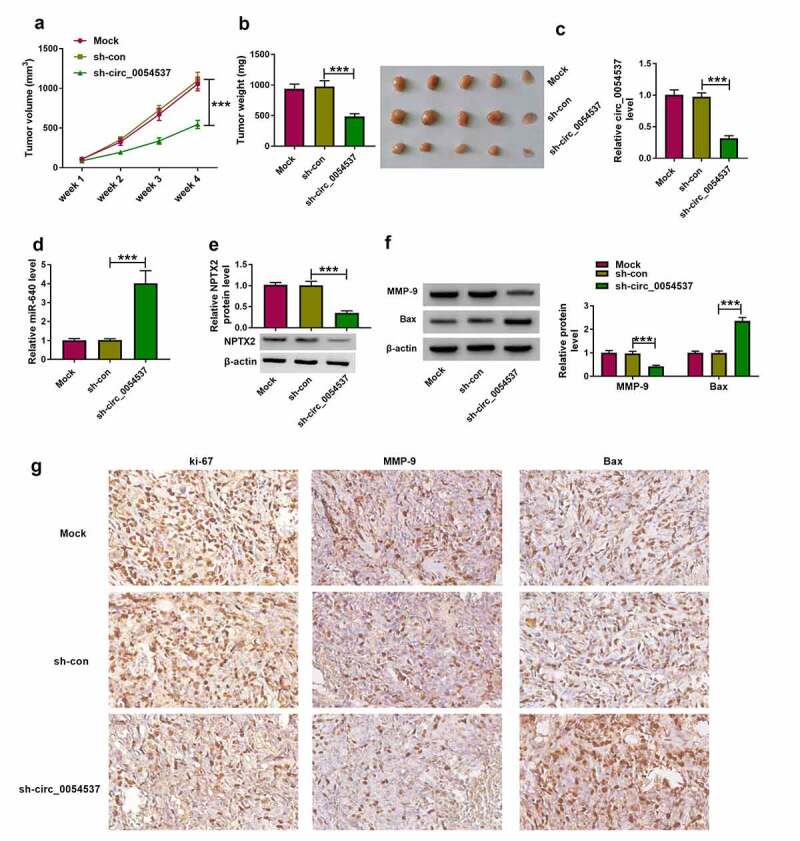
Circ_0054537 deletion retarded tumor growth of RCC in nude mice by regulating miR-640 and NPTX2. (a) Tumor volume in xenograft mice was monitored every week for 4 weeks. (b) Tumor weight was examined, (c, d) RT-qPCR detected circ_0054537 and miR-640 expression, (e) western blotting examined NPTX2 expression, and (f, g) western blotting and/or IHC measured expression of MMP-9, Bax and ki-67 in xenograft mice after 4 weeks. ***P < 0.001

Ultimately, the role of circ_0054537 in regulating RCC cell growth *in vivo* was examined using xenograft experiments. Transfection of sh-circ_0054537 led to a significant decreased in tumor volume and weight, when compared with sh-con group (Figure 8(a) and (b)). Besides, downregulation of circ_0054537 and NPTX2, accompanied with the elevation of miR-640 level in xenograft tumor tissues of sh-circ_0054537 group were found (Figure 8(c–e)). Furthermore, MMP-9 protein level was upregulated while Bax protein level was decreased in xenograft tumor tissues of sh-circ_0054537 group (Figure 8(f) and (g)). Additionally, IHC staining assay uncovered that ki-67- and MMP-9-positive cells were significantly reduced, while Bax-positive cells were increased in xenograft tumor tissues of sh-circ_0054537 group (Figure 8(f) and (g)). These data indicated that circ_0054537 knockdown could suppress RCC cell growth *in vivo* through regulating miR-640/NPTX2 axis.

## Discussion

Given that the five-year survival of patients with malignant RCC is still low, it is still necessary to investigate the pathological mechanism of RCC [34]. Several circRNAs have been disclosed to be closely linked to RCC progression [7]. Here, we investigated the function and mechanism of circ_0054537 in RCC. The *in vitro* and *in vivo* results discovered that circ_0054537 acted as a ceRNA for miR-640 to regulate the expression of NPTX2, thereby regulating cell proliferation, apoptosis, migration, invasion, and glycolysis *in vitro*, as well as mediate RCC tumor growth *in vivo*.

Initially, we found that circ_0054537 was highly expressed in human RCC tissues and cells, was mainly enriched in the cytoplasm. Besides, circ_0054537 possessed a stable circular structure when compared with its host linear gene. A large amount of research has proposed the abnormal expression of circRNAs in RCC, circ_101705 [35], and circ_0039569 [36] were highly expressed in RCC cells, and were strongly linked to CRC cell growth and metastasis. Additionally, Jin et al. has disclosed that circ_0054537 was upregulated in RCC tissues by using circRNA array analysis. They also confirmed that circ_0054537 could promote cell proliferation and migration, but inhibited apoptosis in RCC cells by regulating miR-130a-3p activity [13]. In our research, we also uncovered the reduction of cell proliferation and the elevation of cell apoptosis in circ_0054537-silence RCC cells. Besides, circ_0054537 knockdown inhibited cell migration, invasion, autophagy and glycolysis *in vitro*, as well as to retard tumor growth *in vivo*. These results suggested the tumor-promoter function of circ_0054537 in RCC cell progression.

By searching the microarray data available from GEO database (GSE61741), several miRNAs, especially miR-640, were abnormally expressed in renal cancer tissues. And we found that miR-640 expression in RCC tissues and cells was significantly lower than that in normal tissues and cells. In addition, a negative correlation between miR-640 and circ_0054537 were found in RCC tissues. miR-640 has been suggested to exert a suppressor role in hepatocellular carcinoma [37] and breast cancer [38], whereas its expression and functional effect in RCC remains unknown. Since circ_0054537 distributed throughout the cytoplasm and may act as miRNA sponges, we further confirmed whether circ_0054537 exerts its function in HCC by functioning as miR-640 sponge. Here, miR-640 was predicted as a target of circ_0054537, and was negatively regulated by circ_0054537. Additionally, downregulation of miR-640 partly overturned the anti-tumor effect of si-circ_0054537 in RCC. These outcomes showed a tumor-suppressive role of miR-640 in RCC cell progression.

NPTX2 could serve as an oncogene in RCC, neuroblastoma and colorectal cancer [39–41]. Here, NPTX2 was overexpressed in human RCC tissues, and this upregulation has been been reported previously [20,39,42,43]. Xiang *et al*. [39] further indicated that NPTX2 was associated with overall survival and disease-free survival of RCC patients. Moreover, NPTX2 was proposed as an unrecognized prognostic biomarker and target in human neuroblastoma [40], and a risky methylated gene in glioblastoma [44], even though its methylation was not investigated in RCC yet. However, NPTX2 expression was declared to be specifically increased in ccRCC primary tumors and metastases [20]. In this study, we found that NPTX2 expression was negatively correlated with miR-640, but was negatively correlated with circ_0054537 expression in RCC tumor tissues. Subsequent functional experiments disclosed that NPTX2 was regulated by miR-640 and was involved in the regulation of RCC cell progression by regulating cell proliferation, autophagy, apoptosis, migration, invasion, and glycolysis. Hence, NPTX2 exerted an oncogenic role in RCC cells.

Ultimately, this study suggested that circ_0054537 silencing and miR-640 re-expression might be potential therapeutic approaches to suppress RCC malignancy. Notably, miR-640 was seemed to be firstly identified as an abnormally downregulated miRNA in human RCC, and this study might be the first evidence describing the role of circ_0054537, miR-640 and NPTX1 in aerobic glycolysis and autophagy. Collectively, circ_0054537/miR-640/NPTX1 axis might be a novel molecular mechanism of the pathology of RCC, as well as be a potential pathway for the treatment. However, the limitations of this study were: 1) not further determining the clinical diagnostic and prognostic values of circ_0054537 in RCC, 2) not further probing into the precise mechanism of circ_0054537-miR-640-NPTX2 axis displaying these cellular functions, and 3) not further testing the role of circ_0054537 knockdown in tumor metastasis in nude mice.

## Conclusion

All in all, this study demonstrated that circ_0054537, miR-640 and NPTX2 were aberrantly expressed in human RCC, and silencing circ_0054537 could suppress malignant progression of RCC *in vitro* and *in vivo* by sponging miR-640 to regulate NPTX2 expression. Hence, circ_0054537/miR-640/NPTX2 axis have potential as diagnostic and therapeutic targets in RCC.

## Supplementary Material

Supplemental Material

## Data Availability

All data generated or analysed during this study are included in this published article (and its supplementary information files).

## Highlights

1. Circ_0054537, miR-640, and NPTX2 were abnormally expressed in RCC tissues and cells.
2. Circ_0054537 silence constrained RCC malignant progression *in vitro* and *in vivo*.
3. Circ_0054537 could regulate miR-640/NPTX2 interaction via competing endogenous RNA mechanism.
