# Supplementary material for: Silencing circular RNA circ_0054537 and upregulating microRNA-640 suppress malignant progression of renal cell carcinoma via regulating neuronal pentraxin-2 (NPTX2)
Source: Bioengineered. 2021 Oct 21;12(1):8279–95. doi: 10.1080/21655979.2021.1984002 (PMC8806977; doi:10.1080/21655979.2021.1984002)
Supplement: Supplemental Material [file KBIE_A_1984002_SM7065.zip › supplementary/Supplementary file.docx]

**Cell counting kit (CCK)-8 assay**

Cell viability of 786-O and A498 cells with different transfection was measured by CCK-8 assay. 20 μL CCK-8 solution (Vazyme, Nanjing, China) was added to the medium of each well, and five repeated wells were set up in each group. After incubation for 2 h at 37°C, the optical density (OD) value at 570 nm was measured using an automatic multi-well spectrophotometer (Bio-Rad).

**Transwell assay**

Cell migration ability was evaluated by transwell assay with transwell chamber (6.5 μm pore; Corning, Cambridge, UK). Transfected 786-O and A498 cells were re-suspended in serum-free medium at a density of 1×10^4^ cells/chamber, followed with transferring in the top chamber. Another 400 μL complete medium was placed into the lower chamber. Transwell chambers were maintained at 37°C for 48 h. The migrated cells on the basolateral chamber were fixed with 70% ethanol for 30 min, and stained with 0.2% crystal violet for 30 min at the room temperature. The stained cells were captured under an inverted microscope (Olympus, Tokyo, Japan).

**Flow cytometry (FCM)**

After transfection, apoptotic death of 786-O and A498 cells was evaluated by Annexin-V-fluorescein isothiocyanate (FITC)/propidium iodide (PI) apoptosis detection kit (Vazyme). 1×10^5^ cells were harvested and re-suspended in 500 μL 1×Annexin V Binding Buffer, followed by the incubation of 5 μL Annexin V-FITC and 5 μL PI for 10 min in the dark. Apoptosis rate was regarded as the percentage of cells sorted in Annexin V-FITC+/PI+ and Annexin V-FITC+/PI- quadrants.
