# Supplementary material for: Silencing circular RNA circ_0054537 and upregulating microRNA-640 suppress malignant progression of renal cell carcinoma via regulating neuronal pentraxin-2 (NPTX2)
Source: Bioengineered. 2021 Oct 21;12(1):8279–95. doi: 10.1080/21655979.2021.1984002 (PMC8806977; doi:10.1080/21655979.2021.1984002)
Supplement: Supplemental Material [file KBIE_A_1984002_SM7065.zip › supplementary/Supplementary Table 1.docx]

Supplementary Table 1. Top 10 downregulated miRNAs in RCC bloods based on GSE61741 dataset

| ID | adj.P.Val | P.Value | t | B | logFC |
| --- | --- | --- | --- | --- | --- |
| hsa-miR-34a | 4.45E-17 | 5.25E-20 | -11.1305 | 34.91017 | -4.74433 |
| hsa-miR-640 | 7.45E-13 | 1.76E-15 | -9.2055 | 24.74753 | -4.24002 |
| hsa-miR-516b | 2.78E-08 | 1.64E-10 | -7.0175 | 13.59943 | -3.69887 |
| hsa-miR-604 | 2.30E-12 | 1.08E-14 | -8.8661 | 22.97271 | -3.59129 |
| hsa-miR-214 | 1.49E-12 | 5.27E-15 | -9.0007 | 23.6752 | -3.52219 |
| hsa-miR-891b | 8.59E-08 | 9.11E-10 | -6.672 | 11.93178 | -3.47928 |
| hsa-miR-219-5p | 1.68E-07 | 1.98E-09 | -6.5137 | 11.17975 | -3.11472 |
| hsa-miR-558 | 7.80E-07 | 1.47E-08 | -6.0959 | 9.2361 | -3.08805 |
| hsa-miR-518a-3p | 5.45E-06 | 1.61E-07 | -5.5799 | 6.92807 | -2.9046 |
| hsa-miR-561 | 2.02E-06 | 4.52E-08 | -5.8563 | 8.15075 | -2.90416 |
