# Supplementary figures and images for: Silencing circular RNA circ_0054537 and upregulating microRNA-640 suppress malignant progression of renal cell carcinoma via regulating neuronal pentraxin-2 (NPTX2)
Source: Bioengineered. 2021 Oct 21;12(1):8279–95. doi: 10.1080/21655979.2021.1984002 (PMC8806977; doi:10.1080/21655979.2021.1984002)

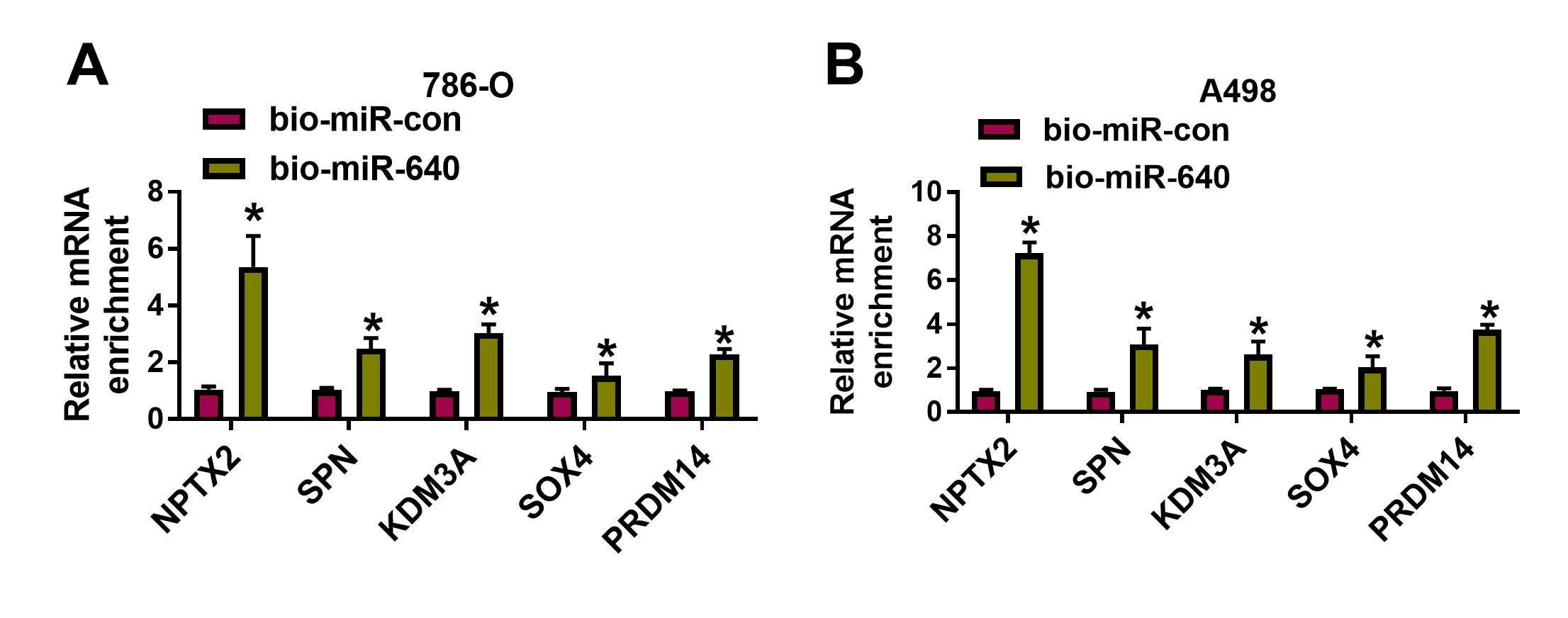

Supplement: Supplemental Material [file KBIE_A_1984002_SM7065.zip › supplementary/Supplementary Fig 1 (1).tif]
